# Supplementary material for: Construction of Hierarchical 2D-3D@3D Zn3In2S6@CdS Photocatalyst for Boosting Degradation of an Azo Dye
Source: Molecules. 2025 Mar 21;30(7):1409. doi: 10.3390/molecules30071409 (PMC11990503; doi:10.3390/molecules30071409)
Supplement: Supplementary file 1 [file molecules-30-01409-s001.zip › molecules-3507556-supplementary.pdf]

Supplementary Information

Construction of Hierarchical 2D-3D@3D $Zn_3In_2S_6$@CdS Photocatalyst for Boosting Degradation of an Azo Dye

Andreas Katsamitros [a], Nikolaos Karamoschos [a], Labrini Sygellou [b], Konstantinos S. Andrikopoulos [b,c], Dimitrios Tasis [a,d,*]

[a] *Department of Chemistry, University of Ioannina, 45110 Ioannina, Greece*

[b] *Foundation of Research and Technology Hellas, Institute of Chemical Engineering Sciences (ICE-HT), P.O. Box 1414, 26504 Rio Patras, Greece*

[c] *Department of Physics, University of Patras, Patras, 26504, Greece*

[d] *University Research Center of Ioannina (URCI), Institute of Materials Science and Computing, Ioannina 45110, Greece*

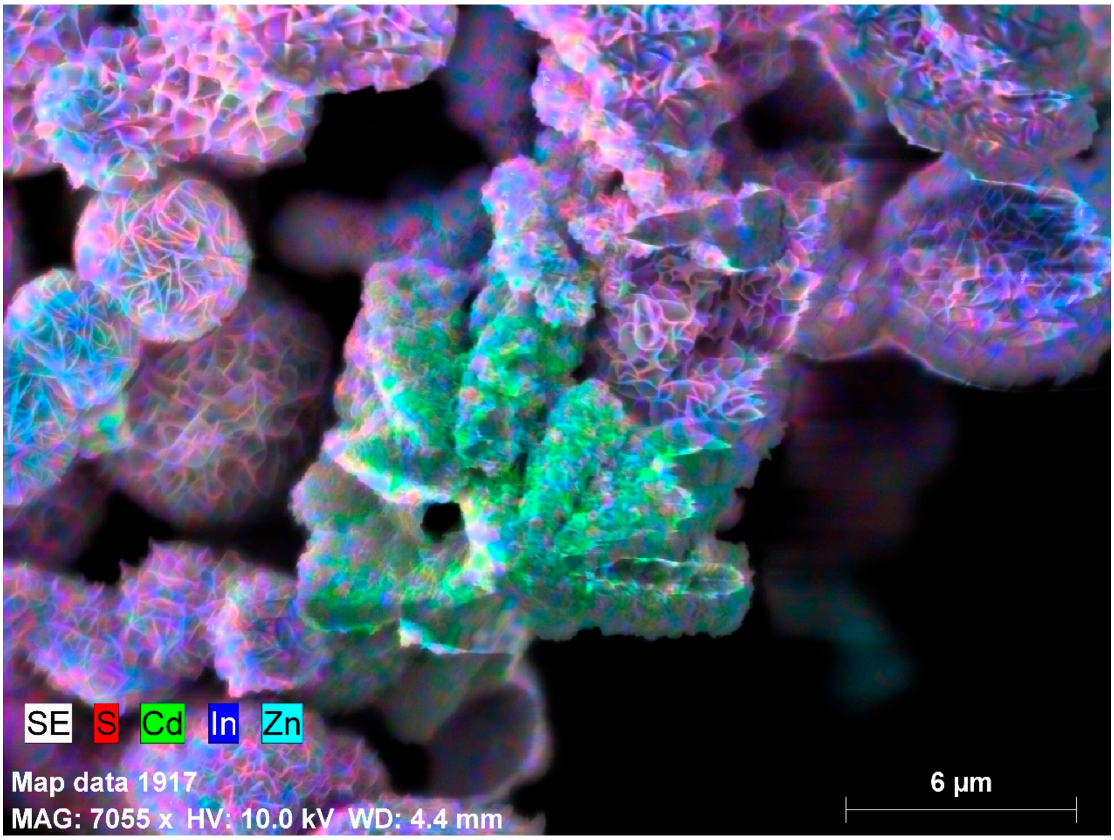

Figure S1. SEM imaging of "ZIS$_3$ 96 wt%" sample by colored elemental domains.

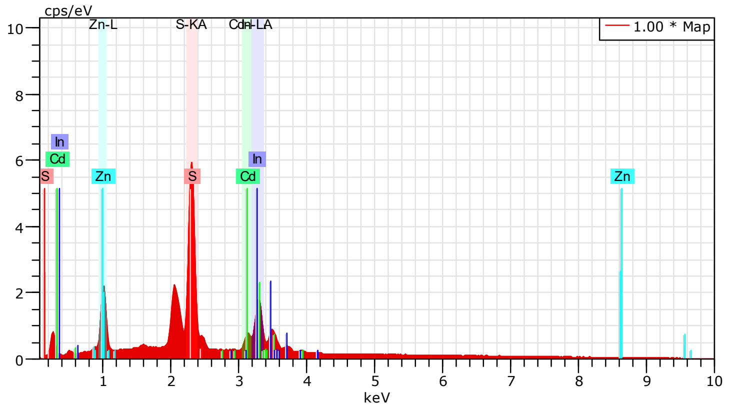

Figure S2. EDX mapping of “ZIS_3 96 wt%” sample.

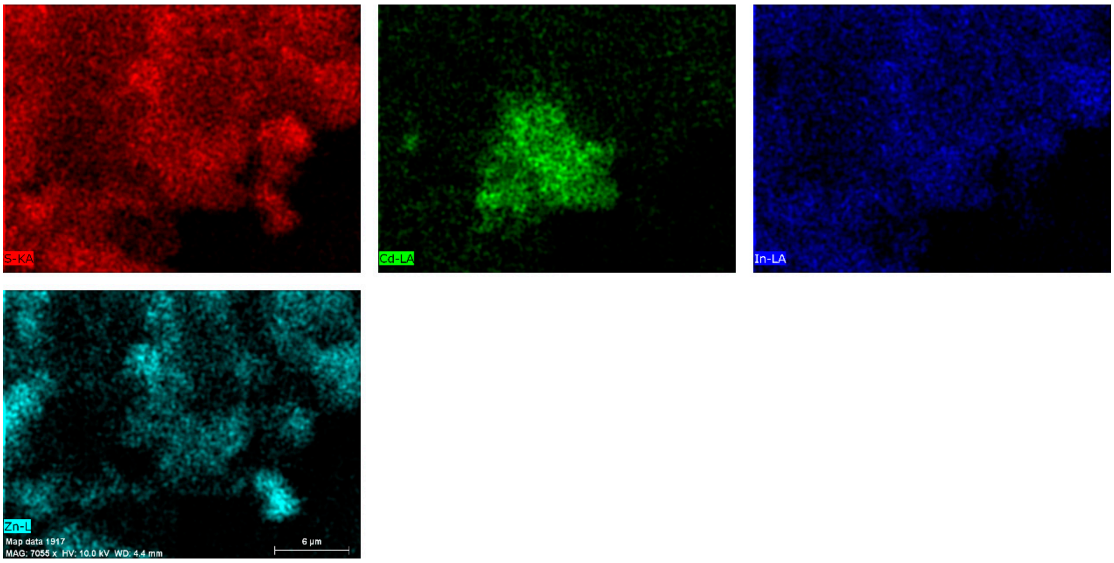

Figure S3. EDX elemental mapping of “ZIS_3 96 wt%” sample.

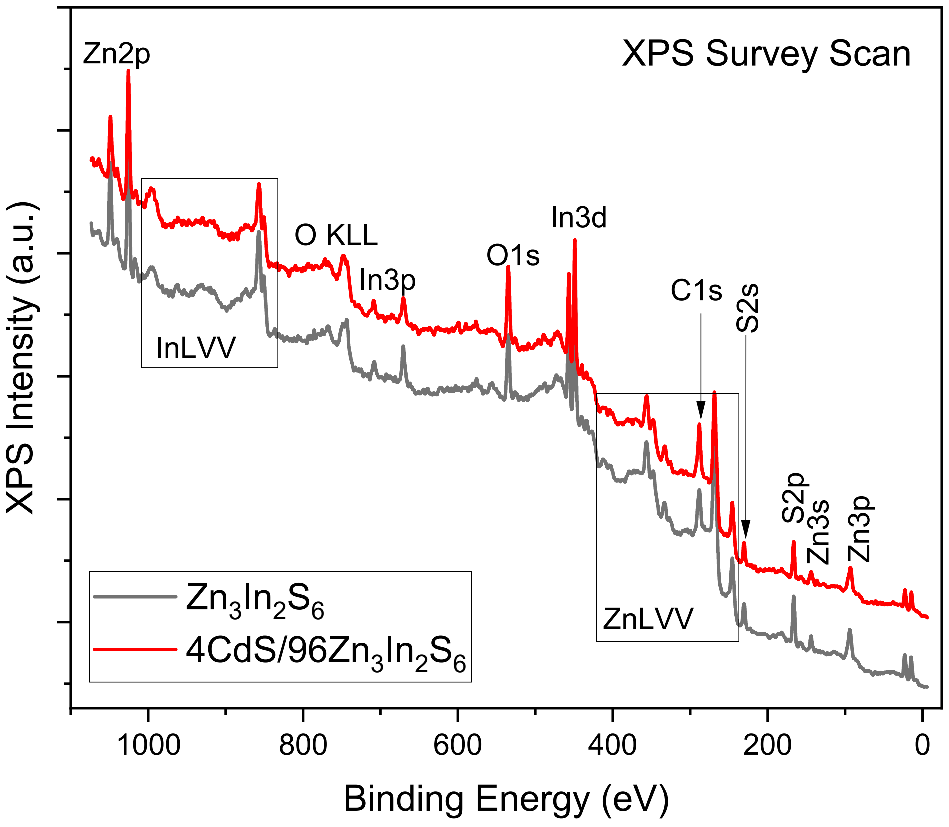

Figure S4. Survey scans of neat $Zn_3In_2S_6$ and "ZIS_3 96 wt%" samples.

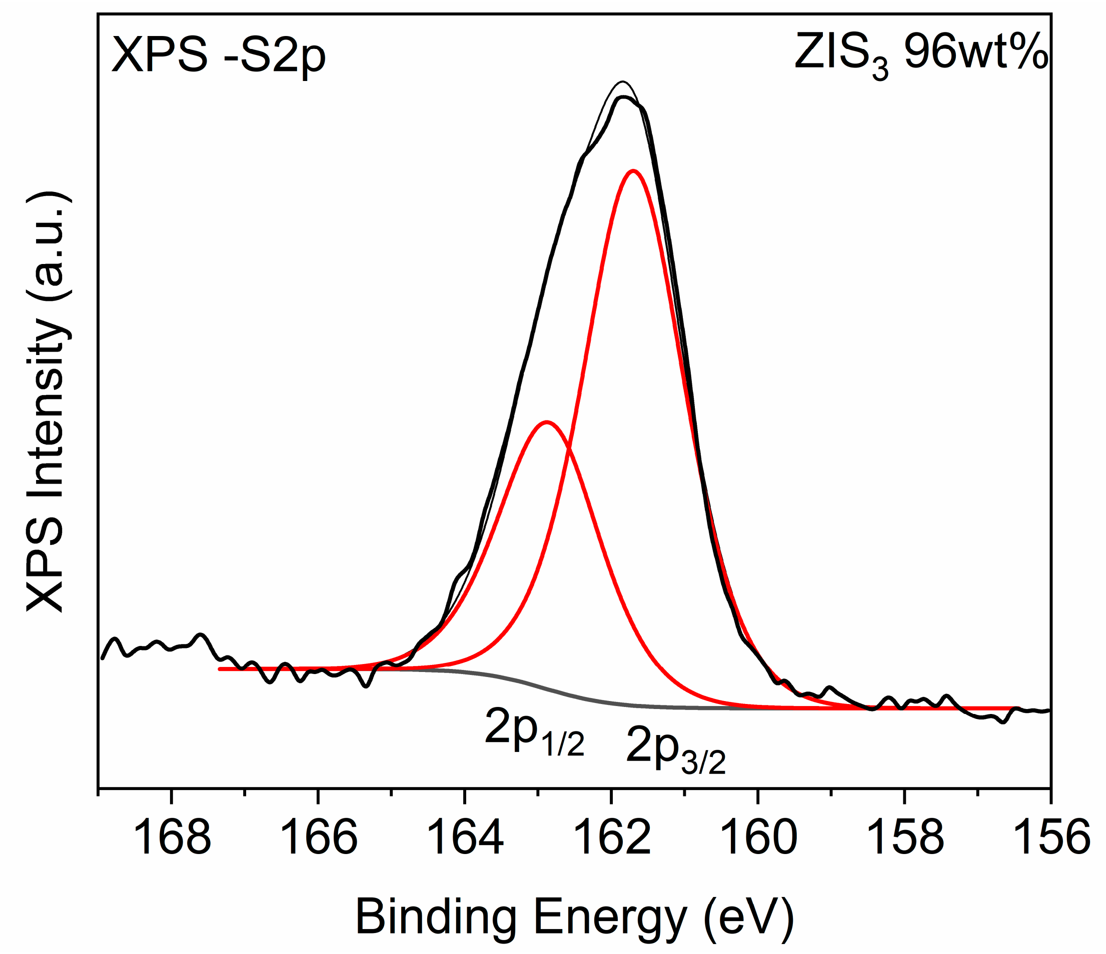

Figure S5. S2p XPS peaks of “ZIS_3 96 wt%” sample.

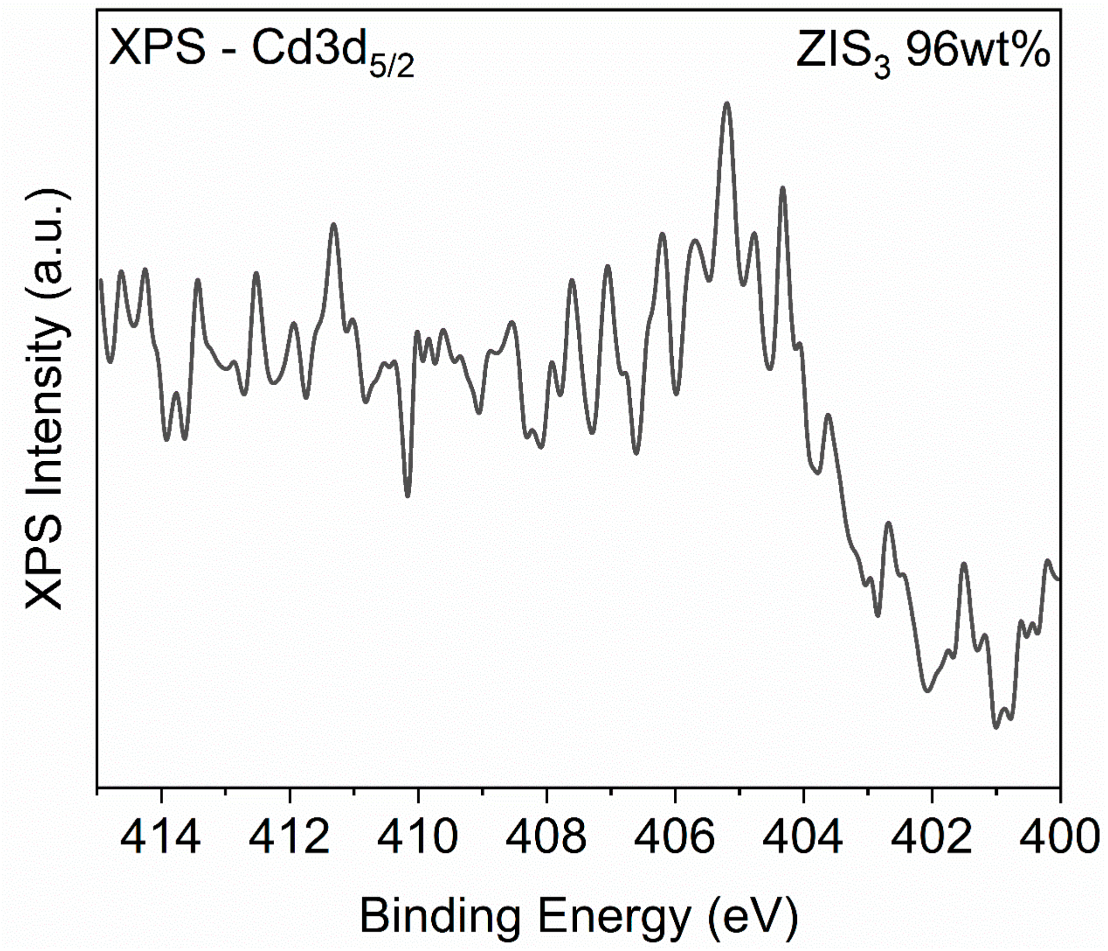

Figure S6. Cd $3d_{5/2}$ profile of “ZIS_3 96 wt%” sample.

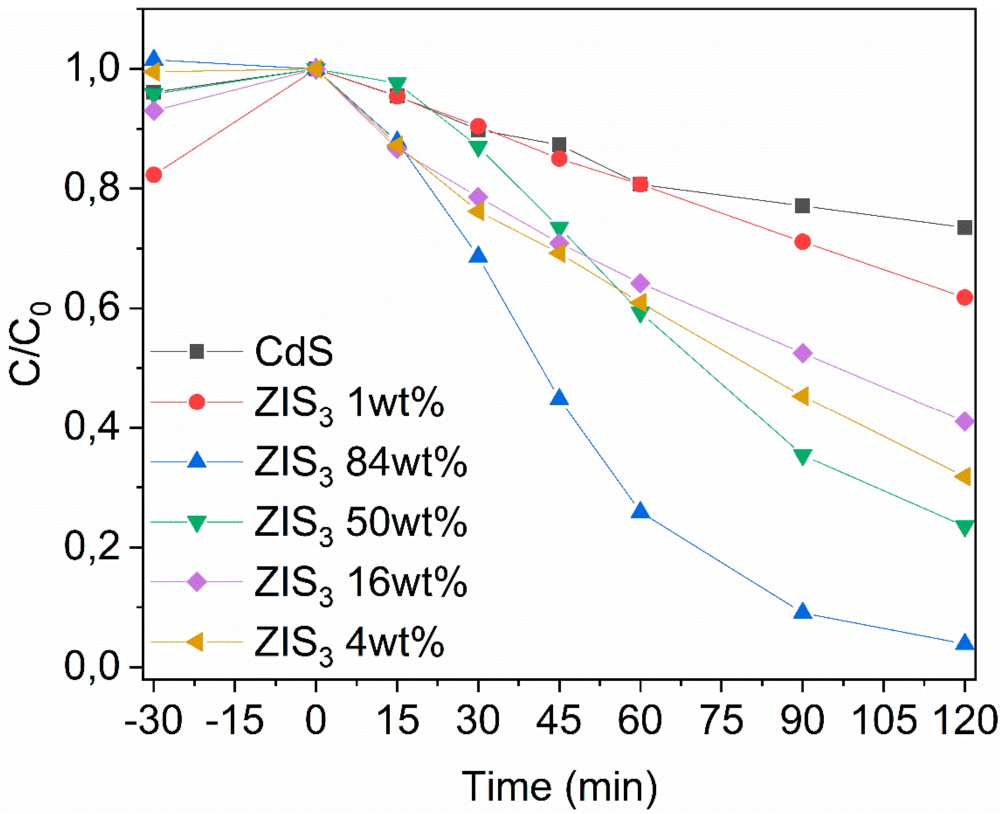

Figure S7. Dye degradation kinetics of various "ZIS_3 x wt%" samples.
